# Factors Influencing Antenatal Care Service Utilization in Hadiya Zone

**DOI:** 10.4314/ejhs.v20i2.69432

**Published:** 2010-07

**Authors:** Zeine Abosse, Mirkuzie Woldie, Shimeles Ololo

**Affiliations:** 1 Hadiya Zone Health Department, Hossana, Ethiopia, P.O. Box 108, e-mail: zeineabosse@yahoo.com; 2 Department of Health Services Management, Jimma University, Jimma, Ethiopia, P.O.Box 1637, e- mail: mirkuzie@yahoo.com

**Keywords:** Antenatal Care, Service Utilization, Southern Ethiopia

## Abstract

**Background:**

In Ethiopia, the levels of maternal and infant morbidity and mortality are among the highest in the world. This is attributed to, among other factors, none use of modern health care services by women in Ethiopia. According to the 2005 Ethiopian Demographic Health Survey, more than seven in ten mothers did not receive antenatal care at all. Therefore, the objective of this study was to explore factors influencing antenatal care services utilization in Southern Ethiopia.

**Methods:**

A community-based cross sectional study was conducted in Hadiya Zone of Southern Ethiopia from January to February 2009. A multi stage sampling technique was used to select the study population in one urban and five rural kebeles. Analysis was done using SPSS for windows version 16.

**Result:**

This study revealed that antenatal care service utilization in the study area was 86.3%. However, from those who attended antenatal care service 406 (68.2%) started antenatal care visit during the second trimester of pregnancy and significant proportion 250 (42%) had less than four visits. Maternal age, husband attitude, family size, maternal education, and perceived morbidity were major predictors of antenatal care service utilization.

**Conclusion:**

Though the antenatal care service utilization is high in the study population, four in ten of the mothers did not have the minimum number of visits recommended by World Health Organization. Promoting information, education and communication in the community is recommended to favorably affect the major predictors of antenatal care service utilization.

## Introduction

Despite progress in some countries, the global number of maternal deaths per year estimated at 529,000 or one every minute during the year 2000 has not changed significantly since the International Conference on Population and Development (ICPD), according to recent estimates by WHO, United nation children's fund (UNICEF) and United Nation fund for population agency (UNFPA) (1). Millions more women survive but suffer from illness and disability related to pregnancy and childbirth (2).

In the case of Ethiopia, the 2005 Ethiopian Demographic Health Survey (EDHS) showed that the maternal mortality rate was 673 deaths per 100,000 live births, and infant mortality rate was 77 deaths per 1000 live births. One explanation for poor health outcomes among women is the non-use of modern health service by a sizable proportion of women in the country (3).

Since long ago, however, it is well known that maternal mortality can be significantly reduced in low-income settings by increasing access to skilled attendants which has close link to ANC, emergency obstetric care and family planning services (4, 5). In spite of this clear importance of maternity care which includes ANC, poor access to and low utilization of such services continue to be important determinants of maternal mortality and morbidity throughout the world (6–8).

Antenatal care is the care given to pregnant women so that they have safe pregnancy and healthy baby. Recently the WHO Technical Working Group has recommended a minimum level of care to be four visits through out the pregnancy. The first visit which is expected to screen and treat anaemia, syphilis, screen for risk factors and medical conditions that can be best dealt with in early pregnancy and initiate prophylaxis if required ( e.g. for anaemia and malaria) is recommended to be held by the end of fourth month. The second, third and fourth visits are scheduled at 24–28, 32 and 36 weeks, respectively (9).

In 2003, analysis of the 2000 EDHS data showed that the use of antenatal care for the most recent birth in the five years preceding the survey was 26.7%. Moreover, there was significant variation of use of antenatal care service by residence. Women from Addis Ababa tended to exhibit the highest use of antenatal care (83.1%), followed by women from other urban (63.4%) and rural areas (21.6%) (10).

Similarly, a study conducted in southern Ethiopia in 2003 showed that the proportion of women who received antenatal care for their most recent birth in the six years preceding the survey was 26.1%. Women living in rural areas were less likely to receive antenatal care than those women in urban areas (3, 11).

This calls for efforts to investigate the critical factors influencing uptake of ANC and other maternal health services. As a result of such efforts a number of socio- demographic characteristics of individual mother affect the underlying tendency to seek care (12). In this regard, good examples are maternal age, education and parity, which have been examined as determinants of health care use repeatedly (10, 13). Another important factor in the utilization of maternity care services, especially in Africa, is the cultural background of the woman (14).

Although this matter has been extensively searched in most parts of the globe, there are few rigorous research efforts to try to explain constraining factors for ANC service utilization among women in Southern Ethiopian. Therefore, this study aimed at exploring factors influencing antenatal care services utilization in the southern region of Ethiopia.

## Methods

A community based cross-sectional study was conducted in Hadiya Zone from January to February 2009. Hadiya Zone is one of the 14 zones in the Southern Nations and Nationalities Regional State of Ethiopia. The zone is administratively divided in to 11 woredas (districts) and 324 kebeles (the smallest administrative unit) and has a population of 1,451,305 about 50% being females. The health services coverage of the zone was 82%. Based on the information from the zonal office, there were one zonal hospital, 17 health centers, 14 upgrading health centers and 244 health posts which all belong to the public health system.

The study area was stratified in to urban and rural areas. In order to select a fairly representative sample the woreda and kebeles were selected randomly. All the ten rural woredas found in the Zone were considered during selection of the study sites. For logistic and cost reasons, three woredas namely Misha, Lemo and Anlemo were randomly selected by lottery method. Similarly, five kebeles (2 from Misha, 2 from Lemo and 1 from Anlemo) were selected. The Capital Town of the Zone, Hossana, was included in the study to represent the urban communities. A total of six kebeles namely; Morsuto and Hage from Misha woreda, Ambichogode and Haise from Lemo woreda, Kebecho from Anlemo woreda and Jalonaramo kebele from Hossana town administration were selected by lottery method.

The study population comprised of mothers who gave birth at least once in the last five years preceding the survey, irrespective of place and outcome of delivery, and who are permanent residents (at least 1 year) of the study area. In cases where a woman had given birth more than once, the most recent pregnancy was considered for the present study. The assumptions during the sample size calculation were: proportion of women attending ANC in the southern region (p=30%), 95% confidence interval, 10% non-response rate, and a design effect of 2. Finally, a total sample size of 710 was reached. The total sample size was then allocated following the rule of proportional to the size of households in each kebele.

A census was conducted in each selected kebele to obtain the list of mothers who gave birth at least once in the last five years. The total number of mothers who gave birth at least once in five years preceding the survey was 1,730. Based on this, a sampling frame which enlists all eligible mothers was prepared and 710 women were randomly selected to be included in the study.

After reviewing relevant literatures, structured questionnaire which meets the objective of the study prepared and part of it was adapted from EDHS 2005 and other similar studies. The contents of the questionnaire include: socio-demographic variables, economic status and ANC service utilization and factors influencing utilization of ANC service. After the instrument was translated into the local language (Hadiyigna) and pre-tested data was collected by ten individuals who completed 12^th^ grade and can speak the local language. A face-to-face interview was conducted going house to house in the selected kebeles. Mothers who are not present during first visit were revisited twice and the result of visiting was recorded on the tool. Two health officers were assigned to supervise and assist the data collection process.

The data from the household survey was entered into SPSS version 16 statistical software. The data was cleaned for inconsistencies and missing values after revision of the original data using the code numbers. Frequencies and summary statistics (mean, standard deviation, and percentage) were used to describe the study population in relation to socio-demographic and other relevant variables. The degree of association between dependent and independent variables were assessed using crude and adjusted odds ratio with 95% confidence interval. Simple logistic regression analysis was performed to assess statistical association between dependent and independent variable and then multiple logistic regressions was also carried out to control potential confounding variables.

Ethical clearance was obtained from the Ethical Review Committee of Jimma University and Southern Ethiopia Regional Health Bureau. Informed verbal consent was obtained from each respondent. Moreover, the right of the respondents to refuse participation was respected and access to the collected data was limited to the research team only.

In this study a mother was put in the category of *‘attended antenatal care’* if she had visited a health facility at least once to receive antenatal care during the last pregnancy.

## Results

A total of 691 women who gave birth at least once within the last five years before the survey were interviewed from one urban and five rural kebeles. The overall response rate was 97.3%. Two hundred forty nine (36%) of the respondents were in the age group of 25–29 with the mean age of 29.38 ± 5.43 ranging from 29.34 ± 5.19 in urban to 29.39 ± 5.48 in rural women. Five hundred forty six (79.0%) and 74 (10.7%) were followers of protestant and Orthodox religions, respectively. Six hundred twenty nine (91.2%) of the study participants were housewives. Eight in ten of the urban and only six in ten of their rural counter-parts had attended formal education. Six hundred forty three (93.1%) of the mothers are currently living with their husband. Three hundred fifty (50.7%) of the respondents earn monthly income of 100-499 Ethiopian Birr (11 Ethiopian Birr=1 USD). Ninety two (78%) of the urban respondents and 454 (79%) of their rural counter parts had family size of greater than five.

The obstetric history of the respondents revealed that 556 (80.5%) of the respondents had their first pregnant before the age of twenty. The mean age at first pregnancy was 19.3 ± 2.5 ranging from 19.0 ± 2.4 in rural to 20.5 ± 2.9 in urban women. Age at first pregnancy was significantly associated with place of residence (p<0.000). Women residing in rural area were 1.5 times more likely to be pregnant before the age of 20 than the urban residents. Out of the total respondents 43 (36.4%) of the urban and 259 (45.2%) of the rural mothers had five or more children. The number of grand multi para was relatively higher among rural than urban respondents. From the total study participants about one in ten of the respondents (11.4%) had lost at least one child before the age of one. Eighty three (12.0%) and 46 (6.7) of the mothers had history of abortion and stillbirth, respectively. In a significant proportion of the respondents (41.5%) the last pregnancy was unplanned.

Among the women included in the study 596 (86.3%) had at least one antenatal visit during their last pregnancy while 95 (13.7%) had none. Out of the total ANC attendants 419 (69.6%) of the mothers received antenatal care from health professionals (doctor, nurse, midwife) for their most recent birth in the five years preceding the survey. While 158 (27.6%) of the mothers received the care from health extension workers (HEW) (minimally trained village health workers) and only 19 (3.2%) of the mothers received care from a trained traditional birth attendant (TTBA) and community health agent (CHA).

Analysis according to residence revealed that 112 (94.9%) of the urban respondents and 490 (84.5%) of their rural counter parts have attended ANC service during their last pregnancy. However, from those who attended ANC service 10.6% of the urban and 16.2% of the rural mothers visited a health institution because they were feeling sick, while 84.2% of the mothers visited a facility for regular check up. Further analysis showed that ANC service utilization is 2 times higher in urban than rural residents (OR = 2.97; 95% CI 1.35, 6.57).

Concerning time of initiating care, only 52 (8.7%) of the ANC attendants initiated care during the first trimester of pregnancy while 407 (68.1%) had the first visit during the third trimester. Number of visit was at least three in 250 (42%) of the ANC service attendants. The majority of the attendants (77.5%) have received 2^nd^ dose of tetanus toxoid during their last pregnancy. However, only 135 (19.2%) and 44 (7%) have received prophylaxis for anemia and drug for intestinal parasites, respectively (Table 1).

**Table 1:** Utilization of ANC services among pregnant women in Southern Ethiopia, 2009

Variable		Residence		
		
		Urban N=118 N (%)	Rural N=573 N (%)	Total N=691 N (%)
**Attended ANC service**				
	Yes	112 (94.9)	484 (84.9)	596 (86.3)
	No	6 (5.1)	89 (15.5)	95 (13.7)
**Person visited**				
	Health professionals	104 (92.9)	315 (64.3)	419 (69.6)
	TTBA/CHA	5 (4.4)	14 (2.8)	19 (3.2)
	HEW	3 (2.7)	155 (32.9)	158 (27.2)
**Reason of visit**				
	Health problem	13 (11.5)	78 (16.2)	91 (15.3)
	For regular check up	100 (88.5)	402 (83.8)	502 (84.7)
**Time of first visit**				
	First trimester	38 (33.6)	15 (3.1)	53 (8.9)
	Second trimester	67 (59.3)	339 (70.3)	406 (68.2)
	Third trimester	8 (7.1)	129 (26.6)	137 (22.9)
**TT vaccination**				
	Yes	105 (92.9)	465 (96.3)	570 (95.7)
	No	8 (7.1)	18 (3.7)	26 (4.3)
**Number of injection (TT)**				
	Single dose	13 (12.3)	116 (24.7)	129 (22.4)
	2nd and +	93 (87.7)	364 (75.3)	447 (77.6)
**Prophylaxis for anemia**				
	Yes	42 (35.6)	93 (16.2)	135 (19.5)
	No	76 (64.4)	480 (83.8)	556 (80.5)
**Drug for intestinal parasite**				
	Yes	10 (12.8)	34 (5.8)	44 (7)
	No	102 (87.2)	450 (94.2)	552 (93)

In this study, maternal age was found to be a factor in the utilization of ANC services. For instance, mothers who are in the age group of 25–29 years were less likely to utilize ANC service than those 35 years and older (OR=0.32; 95%CI 0.16, 0.62). Women whose husbands have positive attitude towards ANC were more likely to utilize ANC than women whose husbands had negative attitude towards ANC (OR=3.5; 95%CI 1.46, 8.34). Family size was found to be a strong factor of antenatal care utilization. Mothers who live in a household having less than three children were eight times more likely to utilize ANC than those living in a household size greater than five (OR=8.14; 95%CI 1.82, 36.44). Mothers with primary educational level were more likely to attend ANC than women who are unable to read and write (OR=0.24; 95%CI 0.14, 0.39). Though residence, marital status (being with husband), occupation and family income have shown significant association during the bivariate analysis and simple logistic regression, multiple logistic regressions has revealed that these variables are not true determinants of ANC utilization (Table 2).

**Table 2:** Socio-demographic determinants of ANC service utilization among mothers in Southern Ethiopia, 2009

variables	ANC utilization		Crude OR	95% CI	Adjusted OR	95% CI

	Yes N (%)	No N (%)				
**Age**						
<20	32 (5.4)	6 (6.3)	0.52	(0.20, 1.34)	0.83	(0.27, 2.53)
20–24	75 (12.6)	6 (6.3)	0.22	(0.09, 0.55)	0.27	(0.09, 0.73)
25–29	230 (38.6)	19 (20.3)	0.23	(0.13, 0.42)	0.32	(0.16, 0.62)
30–34	154 (25.8)	26 (27.4)	0.47	(0.27, 0.81)	0.62	(0.34, 1.15)
>=35	105 (17.6)	38 (40.0)	1 (ref)		1 (ref)*	
**Residence**						
Urban	112 (18.8)	6 (6.3)	0.29	(0.12, 0.68)	0.39	(0.13, 1.18)
Rural	484 (81.2)	89 (93.7)	1		1	
**Ethnicity**						
Hadiya	486 (81.8)	82 (86.3)	1			
Gurage	18 (3.0)	4 (4.2)	1.32	(0.44, 3.99)		
Amhara	32 (5.4)	1 (1.1)	0.18	(0.03, 1.37)		
Kambata	28 (4.7)	3 (3.2)	0.64	(0.19, 2.14)		
Silite	23 (3.9)	3 (3.2)	0.77	(0.23, 2.63)		
Others**	7 (1.2)	2 (2.1)	1.69	(0.35, 8.29)		
**Occupation**						
Housewife	542 (91.1)	87 (91.6)	1			
Civil servant	25 (4.2)	0 (0)	0			
Merchant	15 (2.5)	6 (6.3)	2.49	(0.9, 6.59)		
Student	12 (2.0)	1 (1.1)	0.52	(0.07, 4.04)		
Othersψ	1 (0.2)	1 (1.1)	6.23	(0.39, 100.5)		
**Religion**						
Protestant	474 (79.5)	73 (76.8)	1		1	
Muslim	48 (8.1)	6 (6.3)	0.81	(0.34, 1.96)	0.55	(0.22, 1.40)
Orthodox	66 (11.1)	9 (9.5)	0.88	(0.42, 1.85)	0.68	(0.29, 1.60)
Others§	8 (1.3)	7 (7.4)	5.68	(2.0, 16.14)	4.93	(1.6, 15.19)
**Marital status**						
Married	554 (93.0)	89 (93.7)	1			
Divorced	4 (0.7)	0	0			
Widowed	8 (1.30	1 (1.1)	0.78	(0.09, 6.29)		
Never married	1 (.2)	3 (3.2)	18.67	(1.92, 181.52)		
Separated	29 (4.9)	2 (2.1)	0.43	(0.10, 1.83)		
**Family size**						
1–2	8 (1.3)	5 (5.3)	3.81	(1.21, 11.94)	8.14	(1.82, 36.44)
3–4	119 (20.0)	13 (13.7)	0.67	(0.36, 1.24)	1.13	(0.53, 2.43)
>=5	469 (78.7)	77 (81.1)			1	
**Family income**						
<100	152 (25.5)	24 (25.3)	2.01	(0.97, 4.17)		
100–499	291 (48.8)	59 (62.1)	2.58	(1.35, 4.95)		
>=500	153 (25.7)	12 (12.6)	1			
**Educational status**						
Unable to read &write	159 (26.7)	63 (66.3)	1		1	
Primary school	413 (69.3)	30 (31.5)	0.19	(0.12, 0.31)	0.24	(0.14, 0.39)
Secondary and +	24 (4.0)	2 (2.1)	0.21	(0.05, 0.93)	0.68	(0.13, 3.60)
**Positive husband attitude**						
Yes	171 (96.6)	467 (90.9)	1		1	
No	6 (3.4)	47 (9.1)	2.9	(1.23, 6.98)	1.24	(0.46, 3.32)

* Reference

** Sidama, Tigre, Wolayita, Oromo

ψ Daily labourer, farmer

§ Atheist

Out of the obstetric factors considered, number of delivery experience and parity were found to be determinants of ANC service utilization. As the order of birth decreases utilization of ANC becomes less likely (OR=0.4; 95%CI 0.31, 0.85). Moreover, women whose pregnancy were planned and wanted were more likely to utilize ANC service than those who had unplanned and unwanted pregnancy (OR= 1.76; 95%CI 1.1, 2.8). Mothers who considered pregnancy as a risky event were more likely to seek ANC than those considering it risk free (OR=12.9; 95%CI 7.6, 21.9). It was also found that mothers who are residing with in a nearer walking distance (less than an hour) from a health facility were about 4 times more likely to utilize antenatal care than those residing farther (greater than 2 hours) (OR=3.86; 95%CI 2.08, 7.16) (Table 3).

**Table 3:** Obstetric determinants of ANC service utilization among mothers in Southern Ethiopia, 2009

Variable	ANC utilization		Crude			
	Yes N (%)	No N (%)	OR	95% CI	P – value
**Gravidity**					
1	70 (11.5)	6 (7.2)	0.45	(0.19, 1.1)	0.45
2–4	274 (45.1)	27 (32.5)	0.52	(0.32, 0.86)	0.52
>=5	264 (43.4)	50 (60.2)	1		
**Parity**					
1	76(12.5)	6(7.2)	0.4	(0.31, 0.85)	0.009*
2–4	279 (45.9)	28 (33.7)	0.4	(0.17, 0.98)	0.47
>=5	279 (41.6)	28 (59)	1		
**Ever had abortion**					
Yes	72 (11.8)	11 (13.3)	1.13	(0.57, 2.24)	0.7
No	536 (88.2)	72 (86.7)	1		
**Ever had still birth**					
Yes	37 (6.1)	9 (10.8)	1.87	(0.87, 4.04)	0.10
No	571 (93.9)	74 (89.2)	1		
**Planned pregnancy**					
Yes	364 (59.9)	38 (45.8)	1		
No	244 (40.1)	45 (54.2)	1.76	(1.1, 2.8)	0.01*
**Belief about risk of pregnancy**					
Yes	553 (91.4)	36 (45)	1		
No	52 (8.6)	44 (55)	12.9	(7.6, 21.9)	0.000*
**Walking distance**					
< 1 hour	357 (58.7)	43 (51.8)	1		
1–2 hour	208 (34.2)	20 (24.1)	0.79	(0.45, 1.39)	0.43
>2 hour	43 (7.1)	20 (24.1)	3.86	(2.08, 7.16)	0.000*

**Fig. 1:**
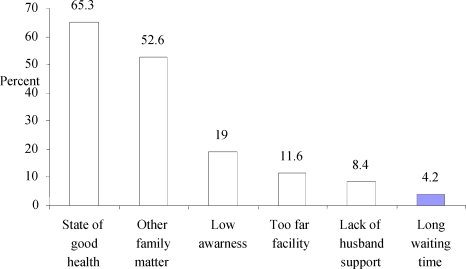
Reasons for ANC non-attendance among mothers in Hadiya Zone, Southern Ethiopia, 2009

Regarding the reason for not attending ANC, 62 (65.3%) of the mothers responded that they were apparently healthy during their last pregnancy. Other reasons mentioned include other family matters, lack of awareness, too far facility, no husband support, and long waiting time (Fig 1).

## Discussion

World Health Organization recommends a minimum of four ANC visits initiated during the first trimester (9). In this study the ANC service utilization rate was found to be 86.3 %. Though this shows a high level of service utilization, more than six in ten of the ANC attendants initiated the visit during the second trimester of pregnancy. Moreover, a significant proportion (41.4%) of the attendees had less than four visits which is less than the recommended.

This finding is consistent with the result of the studies in rural Guatemala and South India which showed 90% and 88% ANC attendance, respectively (15, 16). However, the finding of this study significantly differs with that of EDHS 2005 which showed 30.3% attendance of ANC in the Southern Region of Ethiopia (3). This could be attributed to the fact that DHS covered more remote areas where distance from health institution could be a major predictor of ANC utilization. It is also important to note the time gap between the EDHS and the current study. A study conducted in Northern Ethiopia (2004) showed that the magnitude of ANC attendance was 45% (12).

With regard to the determinants of ANC service utilization; this study revealed that ANC service utilization is significantly influenced by maternal age. Mothers who are in the age group of 25–29 years were less likely to utilize ANC service than women who are 35 years and older. This finding is not consistent with the findings of previous studies conducted in Addis Ababa (1998) (2005) (5, 13).

Positive husband attitude towards ANC was significantly related to antenatal care service utilization. This result agrees with the finding in Addis Ababa (1990) (18). Moreover, in this study the use of antenatal care was found to be related to mother's level of education. Mothers with primary educational level were more likely to attend ANC than women who are unable to read and write. This is in line with other studies conducted in Southern Ethiopia (2003) and EDHS 2005 (3, 11).

It was also observed that availability of women's time is important. In developing countries, women spend more time on their multiple responsibilities for care of children, collecting water or fuel, cooking, cleaning, and trade than on their own health (19). Hence, family size was a strong determinant of antenatal care service utilization. Mothers who live with in a household size less than three people were eight times more likely to utilize ANC than those living in a household size greater than five.

In conclusion, the antenatal care service utilization rate in Hadiya zone is higher than the national figures available to date. However, it is worth noting that majority of the mothers who attend ANC did not receive adequate number of visits and initiated the visits later than recommended by the World Health Organization. Furthermore, maternal education and age, husband attitude, family size and perceived morbidity were major predictors of ANC service utilization. Therefore, efforts to bring about changes in these major predictors at individual and community level through behavioural change communication are recommended.
